# Parameter-Free Binarization and Skeletonization of Fiber Networks from Confocal Image Stacks

**DOI:** 10.1371/journal.pone.0036575

**Published:** 2012-05-14

**Authors:** Patrick Krauss, Claus Metzner, Janina Lange, Nadine Lang, Ben Fabry

**Affiliations:** Department of Physics, Biophysics Group, Friedrich-Alexander University, Erlangen, Germany; University of Jaén, Spain

## Abstract

We present a method to reconstruct a disordered network of thin biopolymers, such as collagen gels, from three-dimensional (3D) image stacks recorded with a confocal microscope. The method is based on a template matching algorithm that simultaneously performs a binarization and skeletonization of the network. The size and intensity pattern of the template is automatically adapted to the input data so that the method is scale invariant and generic. Furthermore, the template matching threshold is iteratively optimized to ensure that the final skeletonized network obeys a universal property of voxelized random line networks, namely, solid-phase voxels have most likely three solid-phase neighbors in a 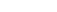 neighborhood. This optimization criterion makes our method free of user-defined parameters and the output exceptionally robust against imaging noise.

## Introduction

**Figure 1:**
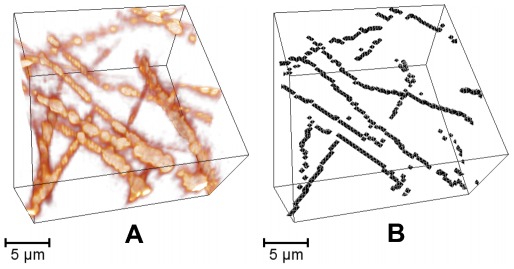
Small 3D stack of a collagen gel Dimensions 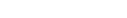 collagen concentration 1.2 mg/ml. (A) Raw data, as recorded with confocal reflection microscopy, without any image processing. The lateral (x-, y-direction) resolution of the fibers is considerably better than the vertical (z-direction) resolution, due to the anisotropic point spread function. In addition, only fiber segments that run in small angles to the imaging plane are visible, due to the so-called blind spot effect. Moreover, the speckled appearance of the collagen network is an optical artifact of reflection microscopy; confocal images of fluorescently labeled collagen networks reveal continuous line networks [16]. (B) Corresponding reconstruction result, using the algorithm described in this paper. Note that voxels that appear to be missing in the reconstruction are located outside of the selected sub volume.

Many biological materials, such as the cytoskeleton or the extracellular matrix, self-organize into complex networks by the polymerization of protein molecules into fibrils (Fig. 1). If the thickness of the fibrils is negligible compared to the pore size, the resulting structure can be mathematically described as a disordered line network. In general, the functional properties of these networks, such as their mechanical stiffness on the macroscopic scale, or their permeability for diffusing particles and for actively migrating cells on a microscopic scale, depend on the geometrical details of the microscopic network structure. In order to study the relationship between structure and function, it is therefore important to extract, or reconstruct, the 3D network structure from image stacks. One aspect of the reconstruction is the binarization of the intensity values of the image stack, so that each voxel is assigned one of two possible values, corresponding either to the solid phase (1, collagen fibers) or the liquid phase (0, surrounding medium). Another aspect of the reconstruction is the skeletonization, so that the optically broadened fibers are reduced to their central (medial) axis, with a width of only one voxel.

While most of the standard reconstruction methods carry out the binarization and skeletonization in a two-step process, our template matching method achieves this in a single step. This new method avoids the problem of choosing an arbitrary intensity threshold for the binarization. Instead, the template matching algorithm automatically adapts the mismatching threshold to the input data such that within the reconstructed fraction of solid-phase voxels the most probable number of next neighbors equals three. This represents a universal property of voxelized line networks.

### Criteria for Reconstruction Methods

We define the following criteria for our reconstruction method: The method needs to be (1) free of user-adjustable parameters, (2) be insensitive to variations in the input data quality and (3) be able to correctly reconstruct known networks. We have applied our method to collagen networks imaged under a wide range of different confocal microscope settings, such as different amplifier gain and laser outlet power. Furthermore, we generated synthetic data sets, with statistical properties almost indistinguishable from measured data but with the advantage that the underlying line network is exactly known.

### Existing Reconstruction Methods

Most of the existing reconstruction methods work with two separate steps of binarization and skeletonization [1]–[6]. The simplest way to binarize an image stack is by comparing each individual voxel’s intensity with a threshold value 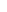 and to assign all voxels that are brighter than 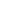 to the solid phase. This method naturally leads to binarized arrays with many artifacts, which need to be corrected in a subsequent step. This correction step includes the simple removal of isolated solid-phase voxels that result from noise. More demanding is the skeletonization which requires the thinning of the broadened binarized fibers to their medial axis of one voxel diameter. Binarization can also lead to the disruption of fibers, so that closing methods, consisting of dilatation with subsequent erosion steps [7], [8], have to be applied as well. Various image processing methods based on convolution kernels, such as Gaussian smoothing filters, Laplace filters or Sobel operators [7], [8] are sometimes used to improve the data quality. However, these methods do not solve the fundamental problem of binarization and suffer from artifacts that have to be removed afterwards.

Another widely used method is template matching [9]–[11], which uses a-priori knowledge of the type of object to be found. Hence, it is possible to compute the degree of similarity between a subvolume of the input stack and a matching template, which is more selective than local or global thresholding with absolute voxel intensities.

Finally, learning algorithms such as vector clustering methods [12] or neural networks [13] have been applied to the reconstruction problem. A significant advantage of these methods is their ability to automatically adapt to the specific properties of the input data. Our proposed template matching algorithm belongs to the class of learning algorithms, since the template is automatically generated from the input data.

**Figure 2:**
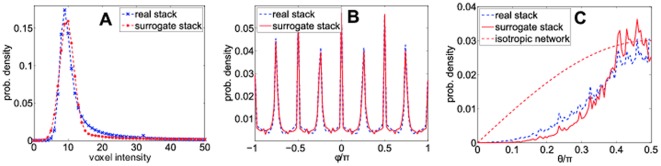
Statistical properties of a real and a surrogate image stacks. (A) Comparison of the voxel intensity distributions in the real and surrogate image stacks. Both distributions are similar. (B) and (C) show angular distributions of the fiber segments. (B) Typical distributions of azimuthal angles 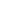 in a real and a surrogate data set. The distributions are almost indistinguishable. The peaks are a result of voxelization. The principal directions, corresponding to the x- and y-direction, as well as the principal diagonals are over-represented in short fiber segments and lead to maxima at 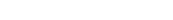 (C) Typical distributions of polar angles 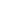 in a real and a surrogate data set. Again, the distributions are similar. Compared to an ideal isotropic network with 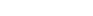, polar angles smaller than 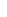 are increasingly suppressed due to the blind spot effect of confocal reflection microscopy [15].

### Limitations of Existing Methods

A detailed summary and comparison of all reconstruction methods is beyond the scope of this paper. Instead, we shall briefly consider the simple example of global threshold binarization and discuss some of its fundamental shortcomings. This will be useful to highlight the advantages of the template matching method proposed later.

We start with an image stack recorded by confocal reflection microscopy. Let us assume that the intensities of the image stack are coded with 8 bits, i.e. all brightness values *B* are in the range [0,255], with *B* = 0 corresponding to completely dark (black) and *B* = 255 to maximum bright (white) voxels. In our setup (Leica SP5X confocal microscope in reflection mode), a typical distribution *p*(*B*) of brightness values has a sharp peak around *B = *15±5 and a flat tail towards large values (Fig. 2A). The reasonable range of binarization thresholds 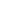 is located somewhere within this tail. However, the distribution *p*(*B*) itself offers no hint for choosing the optimum threshold.

To characterize different network reconstruction methods, we use artificially generated image stacks. This requires realistic models for the network and for its transformation into cross-sectional images by the microscope. As described in more detail in *Methods*, we use a “Mikado” model for the line network, where straight lines of fixed lengths and isotropic orientations are homogeneously distributed throughout the volume [14]. To model the imaging process, we take into account the broadening of fibers (simulated by a convolution with a point spread function), the blind spot effect of confocal reflection microscopy (a gradual darkening of steep fibers) [15] and the addition of random noise. The resulting image stacks have statistical properties almost indistinguishable from measured image stacks (Fig. 2), but with the advantage that the underlying mathematical line network is precisely known.

**Figure 3:**
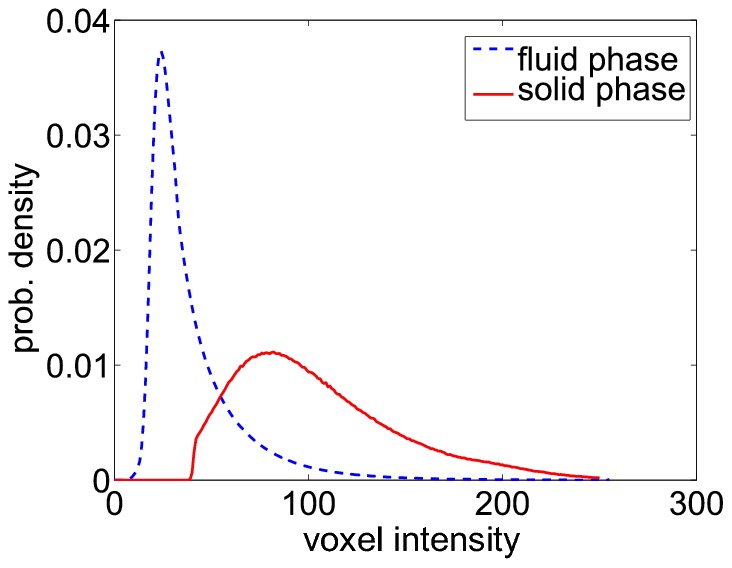
Voxel intensity distributions of the solid and the fluid phase. The two distributions show a wide overlap. No global threshold can be found, even in principle, for separating the two phases, without also producing some false positive and false negative voxels.

To perfectly reconstruct the original line network using global threshold binarization the existence of a threshold 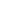 is required, such that all fluid phase voxels have a brightness below this threshold and all solid phase voxels have a brightness above this threshold. However, when we use our synthetic image stacks and plot the brightness distributions of the two phases separately, we find in general two peaks with a significant overlap (Fig. 3). This means that no global threshold can be found, even in principle, for separating the two phases, without also producing some false positive and false negative voxels.

The voxels with brightnesses in the overlap interval include, for example, isolated bright points due to noise. It would be relatively easy to remove them in a subsequent post-processing step. More problematic is that the overlap interval also includes liquid phase voxels from the narrow gaps between two fibers that have been raised in brightness beyond the threshold by the superposition of the fibers’ point spread functions. This effect would lead to a merging of the two close-by fibers in the binarized image and would require a much more sophisticated procedure to be repaired. Finally, the overlap region includes voxels of fiber segments that are more vertically oriented, and are therefore too dark, to exceed the threshold, because of the blind spot effect of confocal reflection microscopy [15]. We note that a human observer could still recognize some of the darker fiber segments in the overlap region quite easily.

Taken together, the threshold binarization has some fundamental limitations. To a certain extent, the method can be improved by using variable thresholds, which take into account the local brightness conditions in the environment of each voxel to be binarized. This, however, can already be viewed as a first step towards a template matching method that will be discussed in the following.

### Template Matching in Line Networks

Template matching methods recognize specific image parts within larger image stacks by comparing features, e.g. the brightness patterns, of small sub volumes of the stack with one or a set of templates. Several recent papers report the tracing of line-like structures, such as actin filaments [9] or microtubuli [10], [11], using 3D template matching. For this purpose, a cylinder segment is varied with respect to size and orientation, resulting in a large set of 3D templates (for example, 850 different orientations in Ref. [11]). In the case of input data with high resolution, due to the large size and number of required templates, this leads to long computation times.

**Figure 4:**
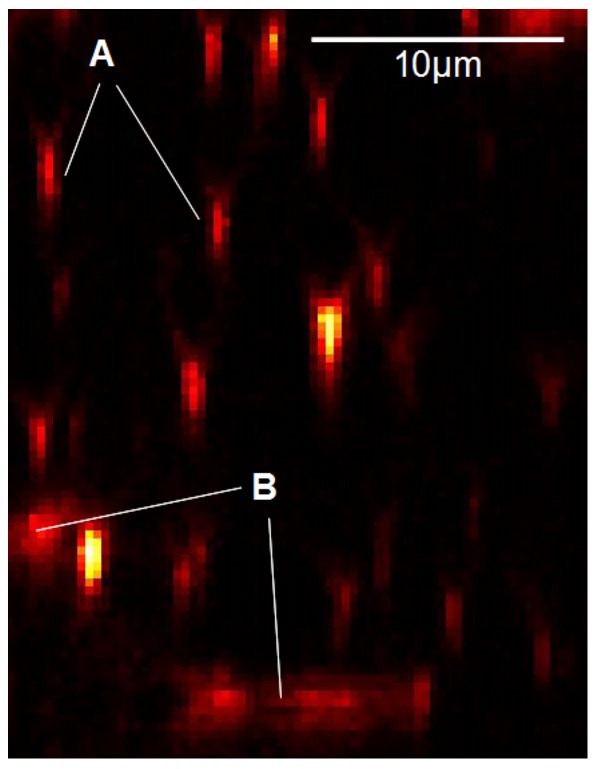
x-z cross-section of a 3D image stack. (A) Perpendicular cross-sections of collagen fibers appear as elliptical spots of finite size that can be easily recognized by 2D template matching. (B) The shape of the spot varies as the angle of intersection becomes less than 90 degrees. For angles less than 45 degrees, the distortion of the spot can become too large to match the template.

However, the situation is much simpler when only 2D cross-sections are used for the template matching: The vertical cross-section of a broadened line segment with a plane is a elliptical spot of finite size that can be easily recognized by 2D template matching (see marking A in Fig. 4). The shape of the spot will vary as the angle of intersection becomes less than 90 degrees. For angles less than 45 degrees, the distortion of the spot can become too large to match the template (see marking B in Fig. 4), but in this case the same line segment can be easily recognized by its intersection with a perpendicular plane. Therefore, all line segments (solid voxels) can be detected by sequentially scanning through the x-, y- and z-direction of the sample volume. As shown below, this binarization method turns out to be much more reliable and robust than the simple threshold method.

We note that this method meets the design criteria imposed before. In order to eliminate all user-adjustable parameters (1), we have implemented an automatic template generator, that is entirely based on the input data. We demonstrate in *Results* that our method is also robust with respect to the quality of the input data (2) and reproduces synthetic line networks almost perfectly (3).

## Methods

**Figure 5:**
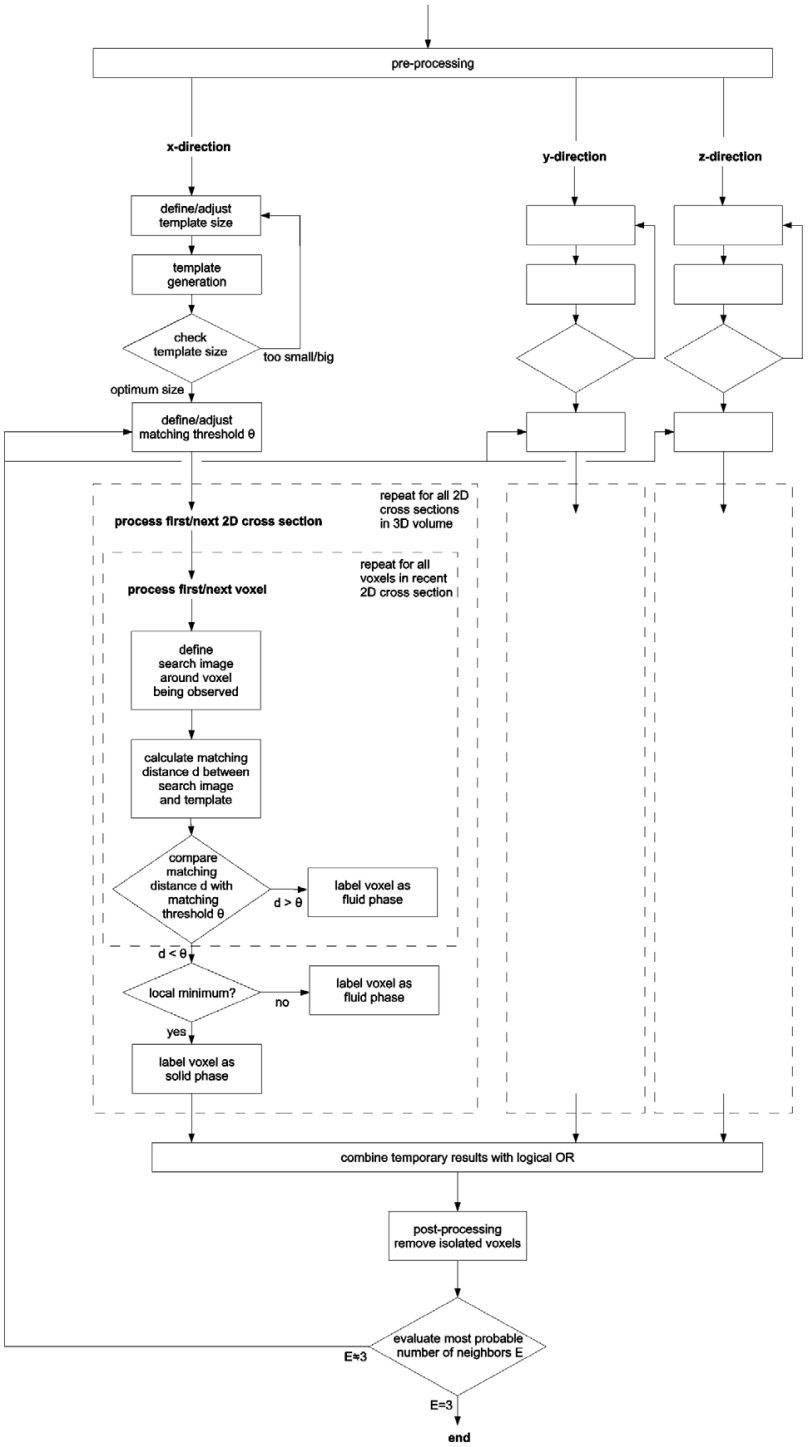
Flow chart of the reconstruction algorithm. The method involves three independent 2D scans through the 3D image stack, along the x-, y- and z-directions. Since these scans are analogous, the diagram focuses on the x-scan only.

The following section outlines the basic methods used in the process of network reconstruction (see also the flow chart in Fig. 5). A more detailed description of the algorithm is available as a preprint: arXiv:1111.3861. In addition, a C++ implementation of the algorithm and a sample data set are freely available at http://tiny.cc/2012-Krauss-PlosOne-Prog.

### Fiber Detection Process

Our method scans through the 3D image stack in x-, y- and z-direction and identifies regions that likely represent sections through a fiber.

Note that our method is generic and applicable to arbitrary network structures and imaging methods. In this report, we focus on collagen gels recorded by confocal reflection microscopy, which does not require fluorescent staining, but leads to a so-called blind spot effect: the apparent brightness of fibers decreases with their angle relative to the imaging plane, leaving all fibers beyond a critical cut-off angle invisible [15]. In this case, it is sufficient to scan only in x- and y-direction.

**Figure 6:**
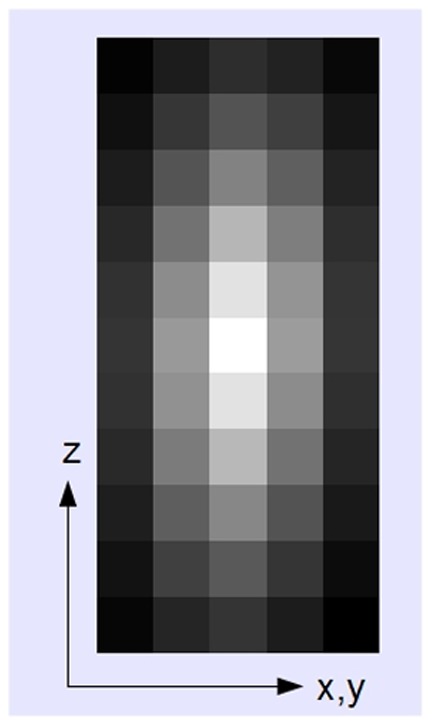
Matching template. Example for an automatically generated matching template in the x-z plane. The blurring is largest in the z direction.

In the following we focus on the scan in the x-direction. The x-scan can be imagined by a y-z-plane (the search plane) that moves through the 3D image stack. The algorithm detects sections of fibers with the search plane by comparing small 2D 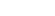-regions of the search plane (the search sections) with a predefined template (Fig. 6) of the same size 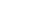. The automatic generation of this template and the optimum choice of its size will be described below. The template, as well as the search sections, are represented as vectors with 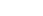 components that correspond to the intensities of the pixels. From all vector components, the global mean intensity of the 3D image stack is subtracted. Finally, all vectors are normalized to magnitude 1 to become independent from absolute intensities. To quantify the mismatch between search section and template we use the Euclidean distance of the corresponding vectors. If this distance exceeds a predefined mismatching threshold (for details see below), the central pixel of the corresponding search section is set to 0 (fluid phase). After this operation, the search plane contains in general numerous localized clusters of solid phase pixels, corresponding to cross sections of individual fibers. Within these clusters, local minima of mismatch (representing the medial axis of fiber cross sections) are determined and set to 1 (solid phase), while all others are set to 0. Analogous scans are performed through the y- and (in case of confocal fluorescence microscopy) z-directions. The binary image stacks resulting from each scan are combined using a logical OR-operation, yielding the final reconstruction result.

### Automatic Template Generation

**Figure 7:**
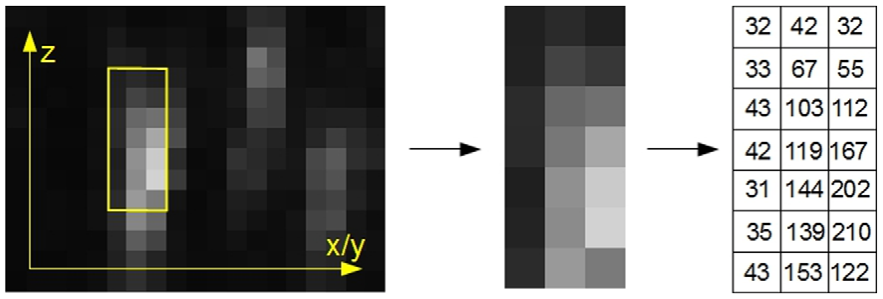
Adaptive template generation. Random sub-sections (yellow window) are selected from the 2D slices of the input stack. They are weighted with the intensity of the central pixel (right side) and then averaged to obtain a representative template for fiber cross sections.

**Figure 8:**
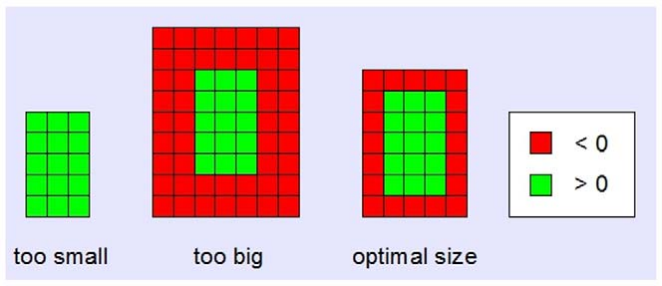
Automatic adaption of template size. After subtracting the mean intensity from each template pixel, the borders of the pattern can be identified by their negative values. Thus, the template can be adjusted to an optimal size. Due to this feature, the algorithm is scale invariant.

The template for each scan direction is generated by weighted averaging over a large amount (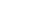) of randomly chosen 2D sections throughout the 3D image stack (Fig. 7). The weighting coefficient of each 2D section is proportional to the intensity of its central pixel. This weighting mechanism assures that only sections that contain a bright fiber at the center contribute significantly to the average. The resulting template represents a typical cross section of a fiber with a bright core and darker borders (Fig. 6). Due to the subtraction of the global mean intensity from each vector component (see above), core pixels are positive, while border pixels are negative. This change of sign is used to automatically adjust the template size (Fig. 8). Due to the automatic size adaption, the algorithm becomes scale invariant with respect to the input data and independent from the optical resolution of the recorded line networks.

### Adjusting the Mismatching Threshold

The choice of the mismatching threshold determines the fraction of voxels labeled as solid phase. For a value that is too low, the reconstructed fibers are disrupted, while a value that is too high leads to thick (not completely skeletonized) fibers. The degree of skeletonization in a voxelized line network can be quantified by counting the number 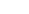 of direct solid neighbor voxels to each solid voxel. By evaluating a large number of voxelized random line networks we determined the distribution function 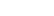 While 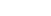 depends slightly on the network density, we find, as a universal property, that 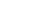 is maximum at 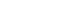 This universal property 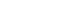 is used to find the optimum value for the mismatching threshold. Our particular choice of 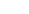 is only valid for line networks, however, and 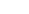 needs to be adjusted in the case of other porous structures to preserve their topology after skeletonization. Needless to say, this requires a-priori knowledge of the network topology.

### Generation of Surrogate Data Sets

For testing the algorithm, we created idealized line networks using a “Mikado” model. Straight lines of fixed lengths and isotropic orientations are distributed with a homogeneous density throughout the volume [14]. Binary surrogate data sets are derived from these parameterized networks by a voxelization operation. We numerically blur these binary data sets to simulate the imaging process and obtain artificial image stacks with gradual intensities. The blurring corresponds to a convolution with an anisotropic Gaussian, representing the point spread function of the imaging system (In the case of confocal reflection microscopy, the gradual darkening of steep fibers with respect to the imaging plane, the so-called blind spot effect, was simulated as well). Uncorrelated image noise was simulated by adding a Gaussian-distributed random value to each voxel intensity. The resulting synthetic image stacks show statistical properties almost identical to measured image stacks (Fig. 2) but with the advantage that the underlying network structure is exactly known.

### Distribution of Nearest Obstacle Distances

Relevant geometric properties of line networks, such as their pore size, are useful parameters to estimate the similarity between different networks. The pore size of a network can be quantified in different ways, for instance by placing within each pore a sphere of the maximum possible size and then analyzing the size distribution of these spheres [16]. In this report we compute the distribution 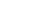 of nearest obstacle distances in the binarized network. This is done by selecting a set of random test points within the stack, computing the distance from each test point to its closest solid state obstacle (i.e. fiber segment) and then finding the distribution of these distances [14], [17].

## Results

In the following sections, we first discuss how voxelization effects may affect subsequent evaluations. Second, we objectively evaluate the accuracy of the reconstruction results by comparing the statistical properties of the original and reconstructed stacks, based on synthetic networks with a known structure. Third, we quantitatively test the robustness of the algorithm with respect to input data quality. Fourth, we demonstrate that our algorithm is invariant with respect to the scale, or resolution, of the input images. Finally, we discuss the computational complexity of the algorithm, its performance on single core computers, and the possibility of parallelization.

### Reconstructed Fiber Networks

**Figure 9:**
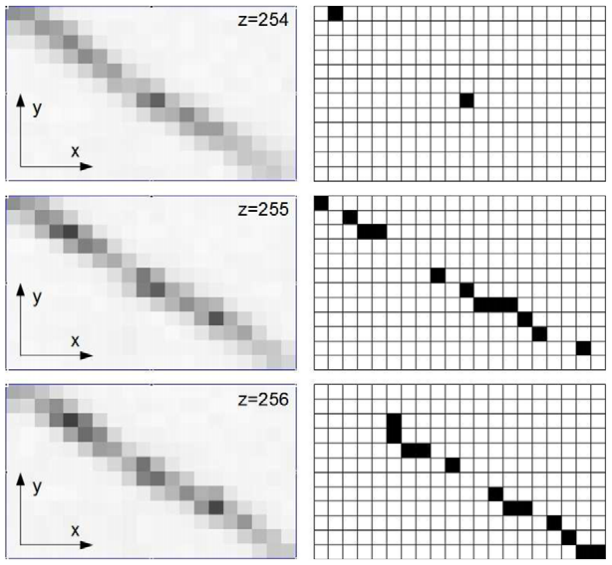
Voxelized representation of collagen fibers. The figure shows three adjacent original confocal images (left) and the corresponding reconstruction result (right). The broadened fiber is reduced to a wiggly, continuous line with a ‘diameter’ of one voxel.

The template matching algorithm reduces the optically blurred images of fibers to contiguous voxel chains of binary value 1 (Fig. 1, see also animation at http://tiny.cc/2012-Krauss-PlosOne-Mov). Within the discrete binary output stack, a fiber that follows a smooth curve in continuous space can only be represented as a wiggling trace of voxels (Fig. 9). This voxelization artifact has no significant effect on the pore size distribution of the line network, however, the analysis of other quantities, such as the local curvature of the fibers, would require further post-processing steps in order to fit smooth space curves through the discrete voxel chains. Alternatively, our template matching algorithm could be extended to sub-voxel accuracy: Once the discrete pixel has been determined in which a fiber intersects a given plane, the detailed sub-pixel position of the fiber’s medial axis in the plane could be found in a second step. This could be achieved, for example, by computing the center of intensity for a small 3D environment around the central voxel.

### Correct Reconstruction of Synthetic Networks

**Figure 10:**
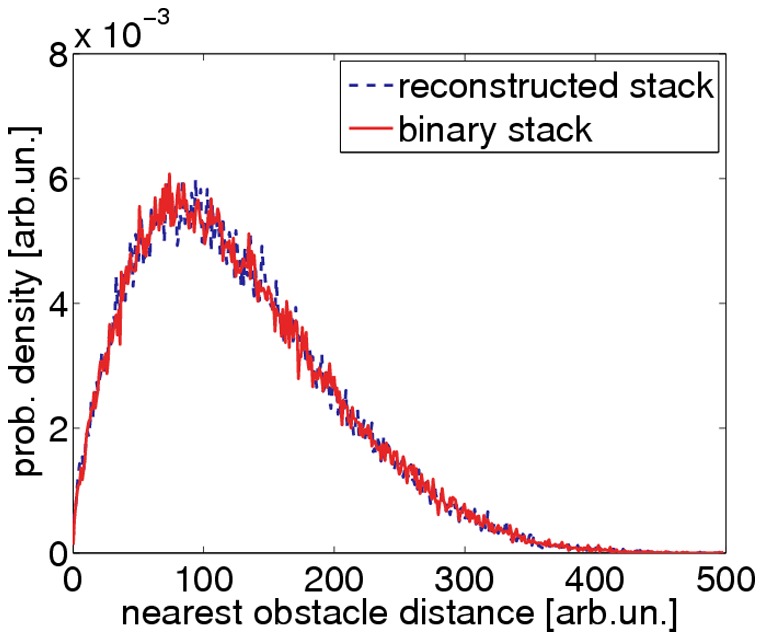
Statistical test of reconstruction quality. We determined the distributions of nearest obstacle distances in a binary surrogate data set and the corresponding reconstruction result. Both distributions are identical, disregarding statistical fluctuations.

The algorithm’s ability to correctly reconstruct random fiber networks was evaluated using surrogate data sets. We generated a set of 100 surrogate image stacks that differed widely in their network densities, point spread functions and noise levels. The quality of the reconstructed networks was evaluated quantitatively by comparing the distributions of nearest obstacle distances in the underlying binary surrogate and in the reconstructed data sets (Fig. 10). The correlation coefficient of corresponding distributions ranged from 0.87 to 0.99, with an average of 0.93.

**Figure 11:**
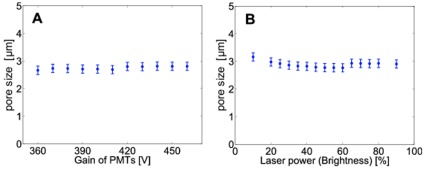
Insensitivity of the algorithm to variations in the input data quality. The algorithm produces stable results over a wide range of photomultiplier gain (A) and laser outlet power (B). Note that the data in (A) and (B) correspond to two collagen gels that have been fabricated under identical conditions. The slight differences in the observed pore sizes reflect sample-to-sample fluctuations.

### Insensitivity to Variations in the Input Data Quality

We performed two tests to evaluate the sensitivity of the algorithm towards variations in the image quality. First, the laser power was kept constant at 3 mW (wavelength 488 nm), while the gain of the photomultiplier tubes was changed over a range of 100 V, corresponding to an intensity variation by a factor of 3.6 (Fig. 11A). Second, the gain was kept constant at a value that gave optimal images for a laser power of 3 mW, while the laser power was varied from 0.6 mW to 5.5 mW, corresponding to an intensity variation by a factor of 5 (Fig. 11B). We find that our binarization algorithm is largely independent of the imaging parameters and the resulting differences in the image quality.

### Scale invariant Reconstruction

**Figure 12:**
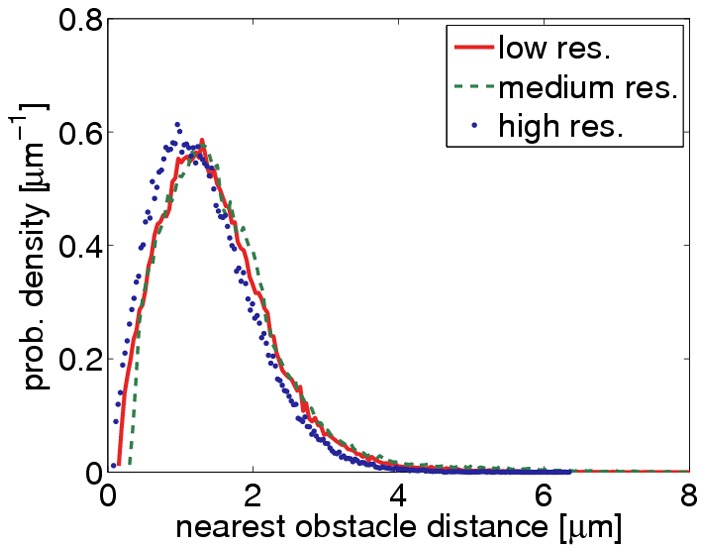
Test of scale invariance. The same collagen gel has been recorded with three different optical resolutions (relative voxel sizes: high/medium/low 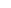 1/2/4). After reconstructing the three image stacks, the distribution of nearest obstacle distances were computed. The low and medium resolutions give similar results. Only at the highest resolution, the pores appear slightly smaller on average, because under these conditions even fine details of the network can be resolved.

Since the templates are adaptively generated from the input data, the algorithm is scale invariant. In order to test this, we recorded the same collagen gel with low resolution (256×256×286 voxels of size 600.6 nm×600.6 nm×168 nm), medium resolution (512×512×512 voxels of size 300.2 nm×300.2 nm×293.7 nm) and with high resolution (1024×1024×1000 voxels of size 150.2 nm×150.2 nm×167.8 nm). The three input stacks were reconstructed and for each binarized stack the distribution of nearest obstacle distances was determined. We found almost identical distributions for the low and medium resolution (Fig. 12). For the highest resolution, the average pore size was slightly smaller because finer structures of the network can be resolved. This trend was confirmed by repeating the experiment with two other gels, including also a dense gel with higher collagen concentration (data not shown).

### Complexity and Performance of the Algorithm

By design, the execution time of our algorithm scales linearly with the total number of voxels in the stack. In C++, the reconstruction of a typical stack (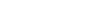 voxels) takes less than 4 minutes on a single core desktop PC, which is an acceptable time for most research labs and is less than the time needed to perform the confocal microscopy measurements. This execution time is to be compared to that of other reconstruction methods, such as [9] (about 1 day for a 1024×1024×512 stack), or [10] (about 70 min. for a 528×256×544 stack, using 54 parallel CPUs). Furthermore, as another advantage of a 2D approach, it would be straight forward to parallelize the algorithm: Since it involves only planar pattern averaging and pattern matching operations, these operations could be performed independently and simultaneously for all the 2D slices in the stack.

## Discussion

In this paper we presented a new method to reconstruct fiber networks from noisy and blurred confocal image stacks. The method is based on template matching across 2D sections of the sample volume, rather than attempting to match 3D fiber segments. Since the algorithm is self-adapting to the specific properties and variable quality of the input image stacks, it does not require any user-defined parameters. In particular, the mismatching threshold is automatically adjusted until the skeletonized fibers have the expected “line-like” property, such that the most probable number of solid phase neighbors to a solid phase voxel equals 3. In addition, the templates are derived from the input data, and the template size is automatically adjusted to the image resolution, so that the method is scale invariant. As a result of the self-adapting properties, the method is robust with respect to imperfections in the confocal image stacks due to varying intensity levels, poor signal-to noise ratio, or strong anisotropic blurring. Finally, we have confirmed the accuracy, robustness and scale invariance of the algorithm using synthetic and real confocal image stacks in which we varied the network geometry, image resolution and image quality over a wide range.
